# Disentangling Bilingualism and Developmental Language Disorder in the Acquisition of Spanish Verbal Agreement Morphology

**DOI:** 10.1017/S0305000926100853

**Published:** 2026-07-07

**Authors:** Patrick D. Thane, Anny P. Castilla-Earls, Ana T. Pérez-Leroux, Alejandra Auza Benavides

**Affiliations:** 1 Department of Spanish and Portuguese, The University of Texas at Austin, Austin, TX, USA; 2 Department of Speech, Language, and Hearing, The University of Texas at Dallas, Dallas, TX, USA; 3 Department of Spanish and Portuguese/Department of Linguistics, University of Toronto, Toronto, ON, Canada; 4 Hospital General Dr. Manuel Gea González, Mexico City, Mexico

**Keywords:** developmental language disorder, bilingualism effects, Spanish as a heritage language, morphosyntactic development

## Abstract

116 bilingual and monolingual children aged 4;0–6;11 with developmental language disorder (DLD) and typical development (TD) completed an elicited production task examining Spanish verbal agreement in the preterit past. Children with TD produced more target-like agreement than peers with DLD. Bilinguals and monolinguals with TD did not differ significantly from one another, but monolinguals with DLD produced more target-like agreement than bilinguals with DLD. Additionally, bilinguals with TD produced more target-like agreement than monolinguals with DLD. Therefore, rates of production of verbal agreement may be useful to distinguish between DLD and TD on quantitative grounds regardless of bilingualism effects. In addition to these quantitative analyses, we document *underspecified forms* (the overextension of tense-marked third person singular and plural) as well as minimal bare forms in children’s production across groups.

## Resumen

116 niños bilingües y monolingües con trastorno de desarrollo del lenguaje (TDL) y desarrollo típico (DT) entre las edades de 4;0 y 6;11 completaron una tarea de producción controlada para examinar la concordancia verbal en el pretérito en español. Los niños con DT produjeron más de la concordancia verbal anticipada que los niños con TDL. Los bilingües y monolingües con DT no se difirieron significativamente entre sí, pero los monolingües con TDL produjeron más de la concordancia anticipada que los bilingües con TDL. Asimismo, los niños bilingües con DT produjeron más casos de la concordancia anticipada que los monolingües con TDL. Por lo tanto, el porcentaje de producción de la concordancia verbal podría resultar útil para distinguir entre el TDL y el DT al nivel cuantitativo independientemente de los efectos del bilingüismo. Aparte de estos análisis cuantitativos, documentamos formas no especificadas (la sobreextensión de la tercera persona singular y plural con morfología de tiempo), así como formas mínimas no flexionadas en la producción de los niños a través de los grupos.

## 1. Introduction

Heritage languages (HLs) are acquired in minority contexts where a majority language has greater social and institutional presence. The increases in exposure to and use of the majority language over time often influence HL development. Research has demonstrated that reduced HL input and crosslinguistic influence from the majority language, which, following Pirvulescu et al. (2014), we address herein as *bilingualism effects*, manifest themselves in consistent ways across HLs. In particular, previous research has shown that bilingualism effects are particularly evident in HSs’ acquisition of inflectional morphology in multiple languages (e.g. Polinsky & Scontras, 2020). To this end, researchers have argued that inflectional morphology is the bottleneck of HL acquisition given the many ways that syntax, semantics, and the lexicon map onto this area of grammar (Montrul, 2018). Spanish, one of the most researched HLs, is an inflected language, while English features poor verbal inflectional paradigms. Therefore, bilingualism effects can significantly shape child Spanish heritage speakers’ (HSs’) morphosyntactic systems.

Relevant to a clinical perspective is the fact that the morphosyntactic innovations that child HSs demonstrate are highly similar to those of children with developmental language disorder (DLD), both bilingual and monolingual. Children with DLD experience unexplained yet persistent difficulties in acquiring all areas of language, but inflectional morphology is a bottleneck for this population as well (Bishop, 2017). Structural similarities between children with DLD and HSs with typical development (TD) can cause the latter group to be misdiagnosed with language disorder. Some estimates indicate that up to 40% of DLD diagnoses in bilingual children are errant, despite underdiagnosis in the general population (Bonuck et al., 2022). Consequently, identifying what constitutes typical bilingual development, what represents DLD, and what is representative of both phenomena (i.e. atypical bilingual development) is critical to identify and diagnose language disorders more appropriately. Establishing ways to distinguish atypical development from TD in Spanish HSs represents Oetting (2018) and Oetting et al.’s (2016) disorder within difference approach, which argues for using tools that are adequately representative of the multilingual context within which bilingual and bidialectal children develop.

We adopt a disorder within difference approach while also evaluating monolingual children, so as to be able to better explore predictors of DLD in all populations regardless of bilingualism effects. Our aim is thus to highlight and validate the ways that Spanish, the language evaluated here, differs systematically between heritage and other varieties while also searching for markers of DLD that are equally reliable for both bilinguals and monolinguals. To date, however, most studies on the acquisition of Spanish morphosyntax have either compared children with DLD to those with TD or have compared bilinguals and monolinguals. Therefore, integrating both approaches side-by-side is essential to appropriately disentangle the effects of language ability and bilingualism that manifest themselves in similar ways in the acquisition of inflectional morphology in bilingual children, such as Spanish HSs in the United States. Our project joins Morgan et al. (2013) and Thane et al. (2025) in comparing language ability and bilingualism in tandem on the acquisition of Spanish morphosyntax, with the aim of more definitively establishing what is (a)typical in the unique context of HL development.

Following Grinstead et al. (2013), we assume that inflectional morphology that is discourse-sensitive is particularly challenging for young children with DLD to master, regardless of whether bilingualism effects are involved. Following these researchers, nominal or verbal agreement inflections that involve the discourse environment are expected to be reliable predictors of DLD. The present study evaluates preterit (perfective past) tense verbal agreement morphology in Spanish in the United States and Mexico to determine if this structure is useful for the identification of a language disorder within these bilingual and monolingual contexts. Verbal agreement suffixes agree with the person and number of the clausal subject. This agreement can be nonlocal, refer to referents outside the sentence, and/or also involve null subjects inferred from the discourse. It therefore is plausibly prone to discourse interface delays in child acquisition and may be a useful predictor of DLD (Grinstead et al., 2013).

In this study, we complement a quantitative analysis of rates of target-like production of verbal agreement morphology with a principled analysis of participants’ alternative responses. Such an approach could identify qualitative differences in response patterns between groups that could also be indicative of DLD independent of bilingualism, which stands to improve diagnostic accuracy. As stated, this study evaluates verbal agreement morphology in the preterit tense. As described later, the preterit is better-positioned than the present simple tense to distinguish between whether particular groups of children produce bare, uninflected verbs (also referenced as minimal forms here), while others default to underspecified forms such as the third person singular that do carry tense inflection. Should children with DLD produce one type of non-target verb (e.g., a bare verb) more than typically-developing peers, such a finding would suggest that this pattern is a qualitative indicator of atypical development that could be useful for diagnosis. We now turn to a principled account of the Spanish verbal system to better explore these possibilities.

## 2. Spanish verbal agreement

All Spanish verbal paradigms for tense, aspect, and mood feature six agreement inflections that together encode person and number (first person singular, first person plural, second person singular, second person plural, third person singular, third person plural).^1^ Verbal agreement morphology licenses null subjects in contexts of discourse stability (Camacho, 2013) and is therefore often essential for sentence comprehension, making it a fundamental area of the morphosyntactic system to master. In contrast, English has weak agreement features that require word-final /s/ with the third person singular in the present simple tense only; there is no agreement morphology elsewhere in the verb system (except with the copular and auxiliary verb *be*). This creates a context for crosslinguistic influence that may affect bilingual development, regardless of language ability: the poor agreement paradigms of English, which generally becomes most children’s dominant language in the United States (see Castilla-Earls et al., 2019, among many others), could result in a levelling of person/number agreement paradigms in Spanish HSs due to bilingualism effects. Moreover, agreement could be challenging for young children with DLD to master because it implies the need for referring to subjects established in the discourse, outside of local boundaries.

### 2.1. Acquisition of verbal agreement

Early research suggested that typically developing monolingual children experience rapid acquisition of Spanish verbal agreement because of the more linear mapping of this agreement onto distinct person/number morphemes and the frequent need to rely on these morphemes to license null subjects. Bel (1998) reported that at age 2;1, monolingual Spanish speakers produce accurately inflected forms at ceiling, at an age when Phillips (1996) reports that English-speaking children do so half as frequently because of its poorer agreement paradigms. However, research has demonstrated distinct patterns in the acquisition of Spanish verbal agreement by children with DLD and those who are bilingual.

Regarding the former group, studies have demonstrated that children with DLD experience difficulty in mastering verbal agreement, although bilinguals and monolinguals have been assessed in isolation. Regarding monolinguals, Bedore and Leonard (2001) documented that in the elicited production of Spanish, children with DLD between ages 3;0 and 5;6 extended third person and singular forms to non-target contexts and produced morphological infinitives more than peers with TD; however, these researchers demonstrated greater precision in verbal inflection in spontaneous production (Bedore & Leonard, 2005). Using elicited production, spontaneous production, and interpretation tasks with monolinguals aged 3;0 to 6;0 with DLD and TD, Grinstead et al. (2009a, 2009b, 2013) similarly demonstrated the extension of the third person singular and morphological infinitives to non-target contexts, particularly in children with DLD. These authors (specifically Grinstead et al., 2013) argue that the challenges for children with DLD who are acquiring Spanish come from the discourse interface. Structures such as verbal agreement, articles, and clitics involve integrating discourse information into morphosyntactic agreement, which makes them challenging to master.^2^ Finally, Castilla-Earls et al. (2020a) found that in their study with children ages 4;0 to 6;9, participants with TD produced more target-like verbal agreement than peers with DLD.

Studies on Spanish HSs with DLD also report difficulty in mastering verbal agreement morphology. Jacobson (2012) reported that English-dominant Spanish HSs with DLD in the upper elementary grades did not produce more target-like verbal agreement than younger peers, despite receiving bilingual education and showing higher production rates of target-like direct object clitics. Additionally, Castilla-Earls et al. (2021b, 2023) reported more target-like production of verbal agreement morphology in bilingual children with TD than peers with DLD. However, these studies do not compare bilingual and monolingual data, an approach that is better positioned to determine what is typical of bilingual development, what represents DLD in all types of speakers, and what may be unique to atypical development of Spanish as an HL (i.e., disorder within difference).

Importantly, however, challenges with verbal agreement are not exclusive to populations with DLD. Multiple studies on child HSs of Spanish have demonstrated that their verbal agreement systems often show similar characteristics to those of children with DLD. The acquisition of verbal agreement by Spanish HSs is influenced by patterns of HL use (Goldin, 2022), and case studies have reported attrition in the early school years (Anderson, 2001; Austin et al., 2021). In particular, Anderson (2001) documented that extension of first and third person singular forms increased over time in the bilingual siblings studied. Studies with older children suggest that attainment of verbal agreement increases by the late elementary years when typically developing children receive bilingual schooling (Fernández-Dobao & Herschensohn, 2021; Jacobson, 2012), however. Recent research with adult HSs has also shown an extension of the third person singular form to first person singular contexts in preterit and present perfect tenses (Camacho, 2022; Giancaspro & Higdon, 2024), similar to studies on DLD in monolinguals. Therefore, bilingualism effects, including crosslinguistic influence from English, the socially dominant language, could account for the loss of Spanish verbal morphology as children enter the school period and begin to receive less exposure to their HL. This could also lead to a period of protracted development whereby older bilingual children and adolescents exhibit quantitatively higher target-like production compared to younger peers at an age range where monolinguals demonstrate mastery of the same structure. Such findings have become increasingly common in research on Spanish (e.g. Cuza & Solano-Escobar, 2025; Corbet & Domínguez, 2020; Thane, 2026, 2025a) and other HLs (e.g. Daskalaki et al., 2023; Jia & Paradis, 2020).

Therefore, while multiple studies have revealed quantitative differences in the production of target-like verbal agreement between children with DLD and TD, more data would be helpful to explore bilingual and monolingual development side-by-side. In our view, the inclusion of heritage and monolingual children is more appropriately able to distinguish typical from atypical development in bilinguals, which is consistent with a disorder within difference approach (e.g. Oetting, 2018; Oetting et al., 2016), while simultaneously highlighting structures that could prove useful in the identification and diagnosis of DLD regardless of bilingualism. This is useful for speech pathologists in clinical settings working with a broader range of Spanish-speaking children, including emergent bilinguals who are functionally monolingual in Spanish but who attend school in an English environment.

### 2.2. Sources of non-target substitutions

As stated previously, Grinstead et al. (2013) argue that verbal agreement as well as other discourse-sensitive structures like articles, clitics, and null subjects (see Dickinson et al., 2023 and Grinstead, 2004) may be harder for children with DLD to master. In instances of discourse integration and delay, it is also prudent to investigate what alternative forms children produce when they do not use target-like inflectional morphology. Such a question is fruitful for multiple reasons. First, in addition to identifying metrics that can distinguish typical from atypical development on quantitative grounds (i.e., rates of target-like production), children’s non-target production patterns may also provide qualitative information that allows us to disentangle disorders within difference. If certain such patterns are unique to populations with DLD, recognizing them can be useful for diagnosis. Secondly, this approach is more holistic and asset-oriented in focusing on what children *do* produce (i.e. alternatives to the target), rather than exclusively on what they do *not* produce (i.e. low rates of target-like production).

Spanish infinitives involve a lexical root, a thematic vowel (most often /a/, sometimes /e/, or least commonly /i/), and an infinitival marker /ɾ/. For instance, the infinitive *hablar* (*to talk*) consists of the verbal root *habl-* (phonetically /abl/), as well as the thematic vowel /a/ and the infinitival marker /ɾ/. Typically, inflected forms apply the relevant tense, aspect, mood, and person/number morphology to this same root as a suffix. The present simple and preterit inflections for Spanish verbs are given in Table 1. Crucially, in the present simple tense, third person singular forms end in the thematic vowel (therefore, they involve the combination of the root and thematic vowel, /a/, /e/, or /i/). For instance, the third person singular of the present tense is *habla* (root */abl/* + thematic vowel */a/*).

**Table 1.** Summary of inflections for present simple and preterit in Spanish verbs affixed to verbal stems Table 1. long description.

Person/number	Infinitives ending in –ar		Infinitives ending in –er/−ir	
	Present simple	Preterit	Present simple	Preterit
First person singular	–o	–é	–o	–í
First person plural	–amos	–amos	–emos/–imos	–imos
Second person singular	–as	–aste	–es	–iste
Second person plural	–an	–aron	–en	–ieron
Third person singular	–a	–ó	–e	–ió
Third person plural	–an	–aron	–en	–ieron

Previous research has documented the overextension of third person singular morphology to contexts that warrant different person/number morphemes by bilingual children (Anderson, 2001; Austin et al., 2021), adult HSs (Camacho, 2022; Giancaspro & Higdon, 2024), and monolingual children with DLD (Grinstead et al., 2009a, 2009b, 2013). One possible explanation is that third person singular represents the bare form (Grinstead et al., 2009a, 2009b, 2013) since Spanish verbal roots are not pronounceable without thematic vowels. Children who produce these forms (i.e. *habla* ) might actually be using a bare form that does not carry person, number or tense marking but that appears to be inflected since it is synonymous with the third person singular. If this is the case, as Grinstead et al. (2009a, 2009b, 2013) and Pratt and Grinstead (2007, 2008) argue, since children who produce such forms might not truly be inflecting for person, number, or tense, they could be involved in an optional infinitive stage in Spanish. This stage may have gone undetected in early research because children appear to produce third person singular forms, crucially in the present tense, that may appear inflected when in fact they are bare forms that replace morphological infinitives. Grinstead and colleagues thus call previous claims of rapid acquisition of verbal agreement into question and argue that many third person singular productions are actually bare forms in the oral production of young monolingual children.

The second possibility is that children produce underspecified forms because they have not yet acquired a stable representation for tense and agreement. Such an account is consistent with Borer and Rohrbacher (2002), who provide similar claims with evidence in English, French, and German. Under such an account, when children have not acquired a particular morphosyntactic feature, they may produce morphologically underspecified forms rather than fully inflected ones and use discourse linking in place of morphosyntactic cues for agreement. Feminine and/or plural categories may result in children defaulting to underspecified forms where one or both features (person and/or number) are dropped. Crucially, children could exhibit a default for person but not number (i.e. the extension of third person singular *and* plural to non-third person contexts) or could produce a form that is underspecified for both categories (i.e., the use of third person singular in first or second person plural contexts). Children may thus produce a particular form that does not show full specification for agreement but that carries tense features. Whether children produce bare forms or underspecified defaults can be nearly impossible to distinguish in the present tense because such forms are homophonous. For this reason, we analyse the preterit past tense in this study. We turn below to the morphophonological nuances of the preterit that enable such a distinction.

### 2.3. The Spanish preterit tense

To date, studies on the acquisition of verbal agreement by children with DLD have either only considered present tense data or have evaluated present simple and preterit morphology side-by-side (e.g., Bedore & Leonard, 2001; Grinstead et al., 2009a, 2009b, 2013). The perfective past tense, known as the preterit, has distinct person/number exponents that allow us to distinguish between bare, uninflected forms and third person singular defaults, yet both tendencies are homophonous in the present. As shown in Table 1, the third person singular inflections of the preterit are –ó and –ió, which do not involve thematic vowels that are also used in infinitive formation. Should children produce forms such as *habló* (third person singular) or *hablaron* (third person plural) rather than *habla* (bare) in the preterit, this would suggest that these children are favouring underspecified forms (tensed third person singular or plural inflections). However, if children produce bare forms like *habla* in the preterit, this argues for minimal forms that do not carry any tense or agreement information. Since underspecified forms and bare forms are homophonous in the present tense, the preterit is well-positioned to tease these possibilities apart.

Crucially, it has not yet been tested whether different groups of speakers (e.g. bilinguals and/or children with DLD) show different patterns of non-target responses in the preterit only. For instance, it is plausible that children with DLD, who have greater difficulties with inflection, would produce bare verbs (e.g. use of bare *habla* instead of preterit *habló* ) at greater rates than children with TD regardless of bilingualism. Relatedly, bilingual children who experience crosslinguistic influence from English, which lacks strong agreement features, could produce more underspecified morphology in the preterit (e.g. frequent use of third person singular *habló* instead of target preterit forms). Consequently, identifying such patterns may be useful on qualitative grounds, which complements quantitative differences of target-like production.

## 3. The study

Our analysis compares differences in production of verbal agreement in children with DLD and TD who are bilingual and monolingual. Such an analysis can elucidate whether this structure is useful for identifying DLD in bilingual populations (i.e. disorder within difference) as well as in monolinguals with atypical development. We also aim to identify the patterns of non-target responses to determine if such patterns are characteristic of atypical development regardless of bilingualism. We address each of these areas in the present study through the following research questions:

1. Do children with DLD and TD produce target-like verbal agreement at different rates?

Based on previous research (e.g. Castilla-Earls et al., 2020a, 2020b, 2021b, 2023; Grinstead et al., 2009a, 2009b, 2013; Jacobson, 2012), we predicted that children with TD would produce target-like verbal agreement more consistently than children with DLD.

1. Do bilingual and monolingual children produce target-like verbal agreement at different rates?

In line with previous research (e.g., Anderson, 2001; Austin et al., 2021; Goldin, 2022), we expected more consistent production of target-like verbal agreement by monolinguals than bilinguals.

1. Do bilingual children with TD produce target-like verbal agreement at different rates than monolinguals with DLD?

Based on previous findings (e.g. Thane et al., 2025), we anticipated that bilingual children with TD would produce more target-like verbal morphology than monolinguals with DLD. Such a result would disentangle the effects of language ability and bilingualism on quantitative grounds in the acquisition of verbal agreement.

1. When children produce non-target responses, do they rely on the bare form (root + thematic vowel) or do they underspecify for agreement?

As there are limited previous data on verbal agreement in the preterit tense, it is challenging to formulate specific hypotheses for this question. Should children produce verbal forms that appear to have third person singular morphology in the present tense (i.e. verbal root + thematic vowel), this would argue for an optional infinitive stage where children do not inflect verbs for tense, person, or number. If children reduce first and second person forms to the third person in the preterit and produce preterit singular forms in the plural, this argues for featural underspecification and consequent overextension of less-specified forms that are inflected for tense. It is important to note that these possibilities could co-occur and may not be mutually exclusive.

### 3.1. Participants

One hundred and sixteen Spanish-speaking children aged 4;0–6;11 (68 boys and 48 girls) participated in this study (average age: 63.6 months, SD: 9.9 months, range: 48–83 months). Sixty-six bilinguals from upstate New York (*n* = 36) and the Houston, Texas (*n* = 30) regions participated in the study: 33 had DLD (BL-DLD group; average age: 57.5 months, SD: 7.0 months, range: 48–82 months) and 33 had TD (BL-TD group; average age 65.1 months, SD: 10.3 months, range: 48–83 months). Bilinguals’ families were from Mexico (30), Puerto Rico (15), Guatemala (one), and the mainland United States (two). Thirteen families reported that none of these was their origin and five did not report any origin. Forty-eight of the 66 children (72%) were eligible for free and reduced-price lunch. Bilingual children’s maternal education level ranged from primary education (20 mothers) to professional or university degrees (20 mothers), with 18 reporting a high school diploma and 4 some university. Maternal education level was not available for four families. Among the bilingual families, 79% of mothers reported speaking only Spanish with their children, 17% reported using English and Spanish, and 2% reported only English use.

Children in the upstate New York region lived in a community without a widespread Spanish-speaking community or frequent representations of Spanish in public spaces, and they attended English-only schools. However, the children from Houston, Texas attended school districts that provided access to transitional or dual language bilingual education. Spanish was more represented in children’s broader communities, although English remained the social majority language of the region. Despite these differences in sociolinguistic circumstances, geographical region was not found to be a significant predictor of morphosyntactic development in previous research involving these data (Castilla-Earls et al., 2021b), so we do not address this variable further.

Additionally, 50 monolinguals from Mexico City, 25 with DLD (ML-DLD group; average age 64.9 months, SD: 8.7 months, range: 50–83 months) and 25 with TD (ML-TD group; average age 68.2 months, SD: 10.5 months, range: 48–81 months), participated in the study. In all, 19 mothers reported receiving primary education, 17 reported high school education, 6 reported some college, and 5 reported a professional or university degree. Maternal education level was not available for three families. These children came from families with low socioeconomic status living in the region of Mexico City.

All children passed a hearing screening and obtained a standard score of 70 or higher on the Kaufman Brief Intelligence Test (KBIT; Kaufman & Kaufman, 2004) nonverbal subscale. Children were classified as DLD based upon at least two criteria to ensure converging evidence. To test for DLD, bilingual children completed the Bilingual English Spanish Assessment (BESA; Peña et al., 2018) morphosyntax subtest in each language (Spanish and English). The BESA, which is normed on bilingual Spanish-speaking children in the United States, yields a cutoff score for diagnosing DLD in the stronger language of 84 for children 4;0–4;11, 85 for children 5;0–5;11, and 81 for children 6;0–6;11. The BESA’s specificity above 90% and sensitivity above 80% result in high diagnostic accuracy for bilingual children. All of these metrics of specificity and sensitivity are considered acceptable following Plante and Vance (1994). Bilingual children were considered to have DLD if their standard score in the stronger language was below the minimum cutoff standard score on this test. In addition, children completed a retell of *Frog, Where Are You* (Mayer, 1980) as a sample of spontaneous production in each language (Spanish and English). Research assistants first narrated the story, and then children retold the story. These spontaneous samples were subsequently transcribed, coded, and checked by trained research assistants and the second author. The percentage of grammatical utterances was extracted from each sample. To be classified as DLD, children’s percentage of grammatical utterances on this spontaneous oral retell task needed to be below 80% in both languages (see Restrepo, 1998 and Simón-Cereijido & Gutiérrez-Clellen, 2007 for similar methods).

Monolingual children completed the *Tamiz de problemas de lenguaje* (TPL; Auza Benavides et al., 2018), which is normed on monolingual Mexican Spanish. The TPL taps elicited production of nominal morphology and features a sentence repetition task. For children aged 4;0–4;11, the TPL has specificity of 83% and sensitivity of 85%. For children aged 5;0–5;11, it has specificity of 81% and sensitivity of 83%. For children aged 6;0–6;11, it has specificity of 81% and sensitivity of 96%. Monolingual children were considered to have DLD if their standard score was below the minimum cutoff standard score on this test. All of these metrics of specificity and sensitivity are considered acceptable following Plante and Vance (1994). Monolingual children completed story retells in Spanish only. The percentage of grammatical utterances in the best language was used as the second criterion for DLD diagnosis (Restrepo, 1998).

## 4. Methods

The analysis of verbal agreement carried out here was assessed using the *Desarrollo Morfosintáctico del Español* (DEME; Castilla-Earls et al., 2021a), an elicited production task that evaluates mastery of multiple areas of inflectional morphology. Note that this task is not standardized and was not used in the identification of DLD but rather served to explore group-level differences regarding the acquisition and mastery of verbal agreement identified in the research questions. The DEME has been used in previous research on the acquisition of morphosyntax by bilingual and monolingual populations (Castilla-Earls et al., 2016, 2020a, 2020b, 2021a, 2021b, 2023; Thane et al., 2025). This task was administered using a booklet, through which trained research assistants elicited morphology using oral prompts that corresponded with pictures on each page. The seven items on the DEME that elicited verbal agreement in the preterit were analysed for this study. One item elicited first person singular, two first person plural, one second person singular, two third person plural, and one third person singular.^3^

We selected only those items in the preterit tense for this project for the reasons outlined previously, precisely because the person/number paradigms in this tense enable a distinction between the production of an inflected but underspecified third person singular verb (see Table 1) and a bare minimal form (lexical root + thematic vowel), which is not possible in the present. None of the inflectional patterns of the preterit tense involve the unmarked form similar to the verb stem that is homophonous with the third person singular in the present simple tense. Therefore, items evaluating preterit morphology were better positioned to allow us to distinguish between the possible sources of non-target productions in children’s response patterns.

## 5. Procedure

Data collection was approved by the Institutional Review Boards of the State University of New York, the University of Houston, and the Hospital General Dr. Manuel Gea González (Mexico City). Children completed the experiment in their school or a location deemed appropriate by caretakers. Prior to participation, all children’s caretakers provided written consent, and children provided assent before participating. Bilingual data collection required three sessions, while monolingual data collection took place during two sessions. The first testing session, common to both the bilingual and monolingual children, was always delivered at the beginning, but the second and third sessions (one in each language) were in random order for bilinguals.

## 6. Results

Data analysis took place in RStudio (R Core Team, 2022) using the *lme4* (Bates et al., 2015), *lmerTest* (Kuznetsova et al., 2017), and *tidyverse* (Wickham et al., 2019) packages. The *broom.mixed* (Bolker & Robinson, 2018), *patchwork* (Pedersen, 2024), and *openxlsx* (Schauberger & Walker, 2014) packages were also used for tidying, summarizing, and reporting the data. Finally, the *here* (Müller, 2020) package was used to maximize reproducibility through relative file paths. Anonymized data were made available along with coding used for analysis on a public GitHub repository (https://github.com/pthane/DEME-Bilingualism-Analysis). Responses to the DEME data were coded and assigned a score of *1* when children produced target-like inflectional morphology or *0* when they contained any other response. This variable allowed for an overall analysis of target-like production. Independent variables for analysis were group (BL-DLD, BL-TD, ML-DLD, ML-TD) and centred age in months.

### 6.1. Quantitative differences in production rates

**Figure 1.**
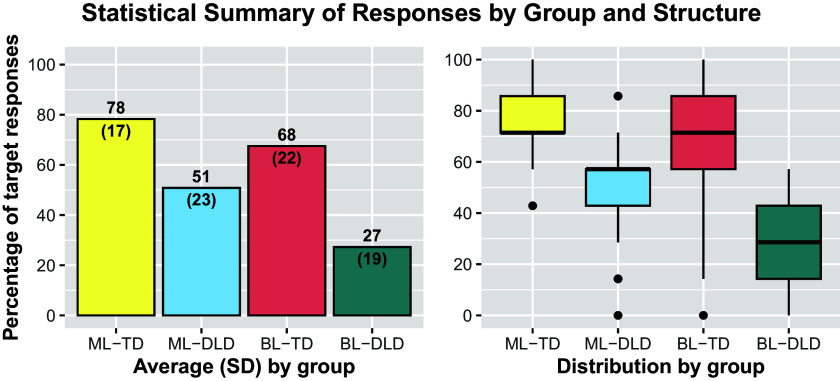
Statistical summary of production of verbal agreement by group. Figure 1. long description.

Each group’s production rates of verbal agreement are shown descriptively in Figure 1; monolinguals produced verbal agreement that matched the person and number of the subject more consistently than bilinguals, as did children with TD compared to those with DLD. Bilinguals with TD outperformed monolinguals with DLD. We note that all groups show individual variability and that no group produced target-like verbal agreement at ceiling.

***Multivariate analysis #1.*** To examine differences in production rates between groups in greater depth, a set of four generalized linear mixed model (GLMM) binomial logistic regressions was necessary with group and centred chronological age in months as predictors. This formed analysis #1. Each group was the reference level for one model (ML-TD, ML-DLD, BL-TD, BL-DLD), enabling group-level comparisons without post-hoc analyses that adjust alpha levels, potentially leading to a type II error. Age was included as a covariate given different average ages across groups. Participant and item were random intercepts in all models. Because chronological age and group do not vary within participants in our cross-sectional data, random slopes for these variables were not feasible to calculate. As data collection took place only once, further random effects structure was not theoretically motivated. Therefore, we included random intercepts only [(1 | Participant) + (1 | Item)], which represented the full nested structure of our data.

We reported the results of these models as the probability of producing a target-like response at an average age of 63.6 months, where a probability of 100% indicates categorically target-like verbal agreement forms and a probability of 0% implies no production of target-like forms. The formula for calculating probabilities, reported here in percentages for ease of interpretation, is prob = (exp(log odds)/[1 + (exp(log-odds))] * 100). The results of these models, including probabilities, are shown in Table 2.

**Table 2.** Results of GLMM models for group and age. Note that probabilities for age in months are reported in 12-month increments. The predicted increase in percentage of target-like responses per 12 months is the probability for age in months subtracted from the intercept Table 2. long description.

Variable	Estimate	Probability	SE	p	Reference group
*Intercept (ML-TD)*	1.40	80.2%	0.42	*< .001*	ML-TD
ML-DLD	−1.40	50.1%	0.32	*< .001*	ML-TD
BL-TD	−0.53	70.5%	0.31	.085	ML-TD
BL-DLD	−2.34	28.1%	0.33	*< .001*	ML-TD
Age in months	0.05	88.0%	0.01	*< .001*	ML-TD
*Intercept (ML-DLD)*	0.00	50.1%	0.40	.994	ML-DLD
ML-TD	1.40	80.2%	0.32	*< .001*	ML-DLD
BL-TD	0.87	70.5%	0.28	*.002*	ML-DLD
BL-DLD	−0.94	28.1%	0.30	*.002*	ML-DLD
Age in months	0.05	64.4%	0.01	*< .001*	ML-DLD
*Intercept (BL-TD)*	0.87	70.5%	0.39	*.026*	BL-TD
ML-TD	0.53	80.2%	0.31	.085	BL-TD
ML-DLD	−0.87	50.1%	0.28	*.002*	BL-TD
BL-DLD	−1.81	28.1%	0.29	*< .001*	BL-TD
Age in months	0.05	81.2%	0.01	*< .001*	BL-TD
*Intercept (BL-DLD)*	−0.94	28.1%	0.40	*.019*	BL-DLD
ML-TD	2.34	80.2%	0.33	*< .001*	BL-DLD
ML-DLD	0.94	50.1%	0.30	*.002*	BL-DLD
BL-TD	1.81	70.5%	0.29	*< .001*	BL-DLD
Age in months	0.05	41.4%	0.01	*< .001*	BL-DLD

These analyses revealed statistically significant differences between all groups at average age with the exception of the contrast between the BL-TD and ML-TD groups. As stated previously, it is critical to point out that all groups produced target-like verbal morphology below ceiling, arguing for a gradual developmental process. Chronological age emerged as significant in all analyses. A one-year increase in age was associated with a 7.8% increase in probability of producing target-like verbal inflection for the ML-TD group, 14.5% for the ML-DLD group, 10.7% for the BL-TD group, and 13.4% for the BL-DLD group.

***Multivariate analysis #2.*** Additionally, we carried out an alternative analysis (analysis #2) by performing Tukey post-hoc comparisons on the model given earlier with ML-TD as the reference level. These comparisons have adjusted alpha levels for four-way comparisons, which decreases the likelihood of type I errors (as opposed to the opposite scenario of undercorrection and type II errors). Converging evidence from analyses #1 and #2 improves the accuracy of reporting effects for particular groups, but diverging evidence across these analyses should be interpreted with caution. The results of these post-hoc comparisons are given in Table 3. The post-hoc comparisons are consistent with the four models in multivariate analysis #1, as all groups differed at the *p* < .05 level from one another with the exception of the two groups of children with TD.

**Table 3.** Results from Tukey post-hoc comparisons for analysis #2 Table 3. long description.

Contrast	Estimate	SE	p
(ML-TD) - (ML-DLD)	1.40	0.32	*< .001*
(ML-TD) - (BL-TD)	0.53	0.31	.311
(ML-TD) - (BL-DLD)	2.34	0.33	*< .001*
(ML-DLD) - (BL-TD)	−0.87	0.28	*.012*
(ML-DLD) - (BL-DLD)	0.94	0.30	*.009*
(BL-TD) - (BL-DLD)	1.81	0.29	*< .001*

### 6.2. Differences in nonfiniteness and substitution

We next turn to a discussion of children’s patterns of non-target use of verbal agreement inflections. To do so, we first selected the 369 (39.2%) responses that did not include target-like verbal agreement morphology. We subsequently coded all 369 non-target responses using a total of ten categories. When children produced the correct person and number features but used the non-target tense (e.g. imperfective past, progressives, present perfect, present simple), their response was tagged as “non-target tense.” Non-target verbal stems that were otherwise correctly inflected were tagged with “stem.” Unintelligible forms were tagged with “unintelligible.” Whenever children did not produce responses, did not use a verb, produced an answer unrelated to the prompt, or used codeswitching within the verb phrase, their response was tagged with “unrelated/no Spanish verb.”

**Figure 2.**
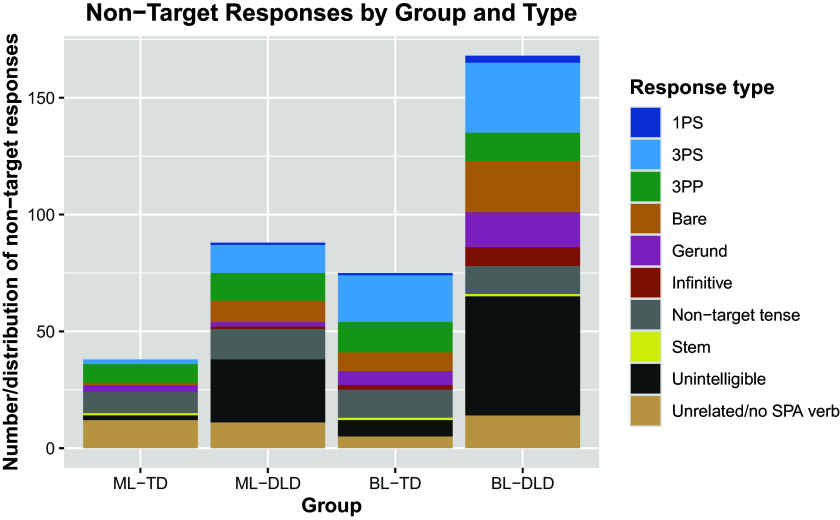
Counts and distribution of non-target responses by group and type. Figure 2. long description.

In addition, six categories were created concerning the use of non-target verbal forms. Whenever a first person singular form was produced in a non-first person singular context, regardless of tense, the tag “1PS” was applied. Similarly, whenever a third person plural form was produced in a non-third person plural context, regardless of tense, the tag “3PP” was applied. There were no first person plural nor second person substitutions in the data, so no tags were necessary for these inflections. Additionally, we applied the tag “3PS” to responses containing an unexpected third person singular form in any tense other than the present simple or present progressive. However, following Grinstead et al. (2009a, 2009b, 2013), a tag of “Bare” was applied to the production of any verb that carried the root and thematic vowel (and therefore appeared to be the third person singular of the present simple or progressive) where another person/number and tense inflection was expected.^4^ Finally, “Infinitive” and “Gerund” were applied wherever infinitival or gerund forms were produced, respectively. Table 4 summarizes the number of non-target forms with each tag by group, which is provided in visual form in Figure 2.

**Table 4.** Number and proportion of non-target responses by group and type. Note that percentages reflect the total within each group, but certain groups may have more non-target responses than others Table 4. long description.

Type of response	ML-TD		ML-DLD		BL-TD		BL-DLD		Total
	#	%	#	%	#	%	#	%	
Stem	1	2.6	0	0.0	1	1.3	1	0.6	3
Unintelligible	2	5.3	27	30.7	7	9.3	51	30.4	87
Unrelated/No SPA verb	12	31.6	11	12.5	5	6.7	14	8.3	42
1PS	0	0.0%	1	1.1%	1	1.30%	3	1.8%	5
3PS	2	5.3%	12	13.6%	20	26.7%	30	17.9%	64
3PP	8	21.1%	12	13.6%	13	17.3%	12	7.1%	45
Bare	1	2.6%	9	10.2%	8	10.7%	22	13.1%	40
Gerund	3	7.9%	2	2.3%	6	8.0%	15	8.9%	26
Infinitive	0	0.0%	1	1.1%	2	2.7%	8	4.8%	11
Non-target tense	9	23.7%	13	14.8%	12	16.0%	12	7.1%	46
Total	38	100%	88	100%	75	100%	168	100%	369

Table 4 and Figure 2 demonstrate that participants with DLD produced a considerable number of responses that were unintelligible. Moreover, across groups, the use of inflected third person singular forms was more frequent than bare forms, and there was also evidence of third person plural and, to a far less frequent degree, first person singular extension to unexpected contexts. To complement these descriptive analyses, we conducted additional statistical modelling to determine whether children who are bilingual and/or who have DLD are more likely to substitute non-target verbal forms than to produce responses without verbs or that are unintelligible. As children with DLD often experience difficulties with areas of language other than morphosyntax, including phonetics and pronunciation (Bishop, 2017), it was plausible that these children would produce a greater proportion of unintelligible responses.

To carry out this analysis, we selected all non-target responses and generated a binary variable called substitution score. We grouped all tags involving the production of verbal morphology (1PS, non-present 3PS, 3PP, bare, gerund, infinitive, non-target tense, and stem) and awarded them a substitution score of 1. This is because these forms comprise verbal substitutions for another form. All unintelligible, unrelated, verbless, or codeswitched responses (subsumed under the “Unintelligible” and “Unrelated/no Spanish verb” categories) received a substitution score of *0*. This is because these responses did not imply substitution of one non-target verbal form for the target-like one.

We then carried out two additional GLMMs with substitution score as the dependent variable and participant and item as random intercepts. In the first model, language ability (TD as reference level, DLD) was the predictor, and in the second model, bilingualism was the predictor (monolingual as reference level, bilingual). Analysing these variables separately allowed us to calculate probabilities of substitution of third person singular forms on the basis of DLD and/or bilingualism distinctly. The results of these models, as well as the probabilities of using a verbal form in non-target responses, are shown in Table 5. These findings suggest that children with DLD are more likely to produce responses that are unintelligible, unrelated, verbless, or codeswitched than children with TD. Children with DLD produced these forms in 37.6% of contexts, compared to children with TD, who did so in only 21% of contexts. However, bilingual children did not differ significantly at the *p* < .05 level from monolingual peers in their likelihood of producing substituted verbal forms rather than unintelligible, unrelated, verbless, or codeswitched responses.

**Table 5.** Results of GLMMs concerning third person singular substitutions Table 5. long description.

Variable	Estimate	Probability	SE	p	Model
*Intercept (TD)*	1.33	79.0%	0.31	*<0.001*	Language ability
DLD	−0.82	62.4%	0.34	*.017*	Language ability
*Intercept (Monolinguals)*	1.00	73.1%	0.25	*<0.001*	Bilingualism
Bilinguals	−0.59	60.2%	0.33	.077	Bilingualism

### 6.3. Inflected versus bare verbs

**Figure 3.**
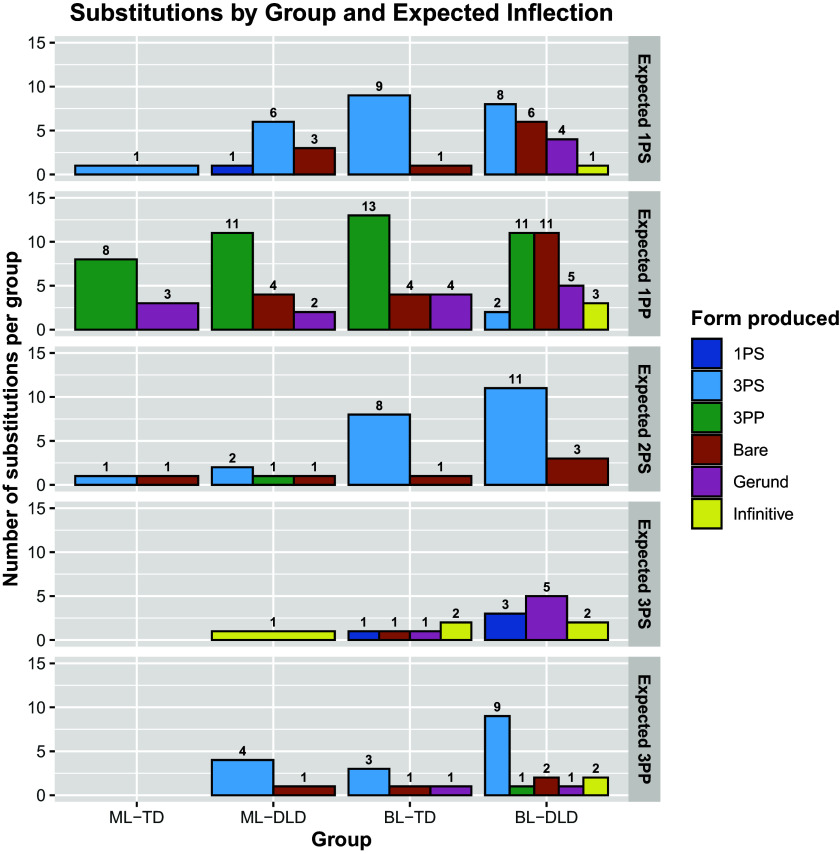
Summary of non-target responses by group and expected form. Figure 3. long description.

Additionally, we aimed to determine if children who have DLD and/or are bilingual differed from typically developing and/or monolingual peers in the extent to which they produced non-target yet underspecified forms versus minimal bare and non-finite verbs. To do so, we further analysed 191 responses with a substitution score of 1 that had the tags of 1PS, non-present 3PS, 3PP, bare, gerund, or infinitive. Figure 3 summarizes the use of each of these forms by group, further subcategorized by which person/number inflection was expected. Descriptively, the suppliance of underspecified forms that carry past tense third person morphology, both singular and plural, is documented more consistently than the production of minimal bare verbs, although both patterns occur in the data. Crucially, third person singular forms with past tense morphology emerged in first and second person contexts, but seldom vice versa; moreover, third person plural morphology occurred in expectedly first person plural contexts, but never vice versa. Substitution is infrequent in third person contexts, arguing for the overextension of underspecified third person forms. The data thus suggest that there are correctly inflected forms, underspecified forms that drop person and/or number features and resort to third person morphology, and minimal bare verbs that do not carry inflection in the data for all groups.

To determine whether children were more likely to produce inflected versus non-inflected forms, we derived an additional variable that we called finiteness score. Responses tagged with 1PS, 3PS, and 3PP were awarded a finiteness score of *1* because they included inflected forms, even though these forms did not match the person and number of the subject. Responses tagged with Bare, Gerund, and Infinitive were awarded a finiteness score of 0 because they were minimal and non-finite. Subsequently, we carried out two final GLMM models with finiteness score as the dependent variable and participant and item as random intercepts. As in the previous analysis, the first model had language ability as the predictor (TD as reference level, DLD) and the second had bilingualism as the predictor (monolingual as reference level, bilingual). As in the previous set of models, analysing these variables separately facilitated the tabulation of probabilities of producing inflected defaults rather than uninflected forms. The results of these models are given in Table 6. Children with DLD were not more likely to produce inflected forms than children with TD at the *p* < .05 level, nor were bilinguals compared to monolinguals. Therefore, finiteness does not appear to hold promise in distinguishing between typical and atypical development when analysing non-target production of verbal forms.

**Table 6.** Results of GLMMs concerning verb finiteness in non-target production Table 6. long description.

Variable	Estimate	Probability	SE	p	Model
*Intercept (TD)*	0.15	53.7%	0.48	.758	Language ability
DLD	0.91	74.3%	0.50	.066	Language ability
*Intercept (Monolinguals)*	0.26	56.4%	0.46	.580	Bilingualism
Bilinguals	0.97	77.4%	0.59	.098	Bilingualism

### 6.4. Individual differences

**Figure 4.**
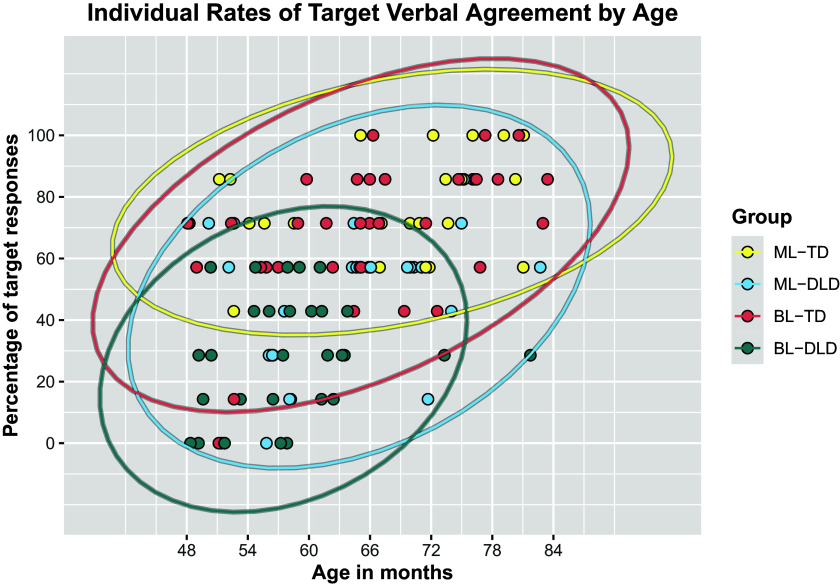
Individual rates of production of target-like verbal agreement. Figure 4. long description.

Finally, we conducted an analysis of individual participants’ production of verbal agreement. Each participant’s percentage of target-like verbal agreement morphology is summarized in Figure 4, organized by group. The ellipses on this graph encompass 95% of all participants within each corresponding group. These data provide three additional observations not accounted for in the group-level analyses. First and foremost, typical children achieved 100% mastery of verbal agreement as early as 64 months, both in monolingual and bilingual contexts, when using the DEME elicited production task here; however, none of the children with DLD produced target-like verbal agreement in 100% of contexts. Secondly, and relatedly, all children older than 58 months produced at least one instance of target-like verbal agreement, but there were children from the BL-TD, BL-DLD, and ML-DLD groups who did so younger than this age. These two findings suggest that the majority of participants in all groups alternate between target-like and non-target morphology or are using this structure at ceiling. Thirdly, we highlight that the four circles overlap between 40% and 60% target-like production and up to 75 months of age, suggesting that there is some individual variation within each group that resembles that of other groups. This is a key finding because it highlights that while group-level trends are critical in accounting for the data observed, some individuals appear similar to peers in each of the other four groups in their production of verbal agreement. We return to a broader discussion of how best to account for these individual differences as an opportunity for future research in our discussion.

### 6.5. Summary

To summarize, verbal agreement distinguished between children with TD and DLD regardless of bilingualism; however, typical bilinguals and monolinguals did not differ at the *p* < .05 level in their target-like production of preterit verbal agreement morphology. The ML-DLD group produced more target-like verbal agreement than the BL-DLD group, but less than the BL-TD group. Children with DLD were often unintelligible, but non-responses, verb omission, and use of English or codeswitching were rare. Children frequently substituted third person singular and, to a lesser degree, third person plural morphology in unexpected contexts, and produced lower rates of bare verbs, gerunds, and morphological infinitives. However, the use of non-target default forms versus bare and uninflected forms did not differ significantly at the *p* < .05 level between children with DLD and TD nor between bilinguals and monolinguals. The individual analyses highlight that most participants vary between target-like and non-target verbal agreement, and there is considerable variation within each of the groups that cannot be captured through group-level analyses alone. We turn to a broader discussion of these results relative to our research questions in our discussion.

## 7. Discussion

Previous research has demonstrated that Spanish HSs and Spanish-speaking children with DLD present similar morphosyntactic characteristics to one another, and that structures involving the integration of discourse such as person/number verbal agreement may be particularly challenging to acquire by children with atypical development. Studies comparing bilinguals and monolinguals with both DLD and TD position us to establish what is typical within TD in a context of HL acquisition (i.e. difference), what may be an indicator of DLD (i.e. disorder), and crucially, what represents atypical development within bilingual varieties (i.e. disorder within difference; Oetting, 2018; Oetting et al., 2016). Our project differed from studies typical of this framework because it also included monolingual children with the goal of pinpointing structures that can be used as clinical indicators of DLD in Spanish-speaking children regardless of bilingualism effects. We incorporated four research questions to broaden our understanding of children’s acquisition of verbal agreement in heritage and monolingual Spanish. We considered the Spanish preterit given its potential to adjudicate between underspecified forms and minimal bare verbs in children’s non-target production patterns (see Table 1).

Our first research question concerned differences in the production of target-like preterit verbal agreement between groups of children with DLD and TD. Children with TD exhibited higher target-like production rates, which supported the hypothesis. This finding aligns with the extant research that has tested bilinguals or monolinguals separately (e.g. Bedore & Leonard, 2001, 2005; Castilla-Earls et al., 2020a, 2023; Grinstead et al., 2009a, 2009b, 2013; Jacobson, 2012).

Similarly, our second research question addressed differences between groups of bilingual and monolingual children. Contra our hypothesis, the BL-TD and ML-TD groups did not differ significantly in the statistical modelling, despite descriptive differences (see Figure 1). However, children in the ML-DLD group produced significantly more target verbal agreement than peers in the BL-DLD group. Therefore, these results, together with the positive effect of age, do not point to the attrition of verbal agreement as has been found in previous studies with a similar age range (Anderson, 2001; Austin et al., 2021; Goldin, 2022). BL-TD and ML-TD children do not differ statistically at the group level, although the former group shows greater individual variation (see Figure 4). It is important to point out that children in the ML-TD group also exhibited variability between target and non-target use of verbal agreement morphology, indicating that mastery of the verb system is still ongoing in the early school years. Therefore, it cannot be claimed that bilinguals’ use of non-target verbal inflection is exclusively due to contact with English, which has a less specified agreement system. Findings lend support to Grinstead et al.’s (2013) proposal that inflectional structures involving the discourse interface are challenging to master and are vulnerable in contexts of atypical development, as in the present study. In fact, this approach appeared to apply to all groups of children tested here, to differing degrees.

Our third research question addressed whether there were differences between the BL-TD and ML-DLD groups in their rates of target-like production of verbal agreement. This question is important for determining the feasibility of verbal agreement as useful for identifying DLD in bilingual populations. We anticipated that the BL-TD group would produce more target-like verbal agreement than the ML-DLD group, which was supported through our findings. Therefore, on quantitative grounds, verbal agreement morphology appears to be a reliable structure for distinguishing between what is typical of bilingualism and what comprises a clinical need because both groups of children with TD produced more target-like verbal agreement than peers with DLD. Our findings join Thane et al. (2025), who found that clitics, but not articles, distinguished between children with DLD and TD regardless of bilingualism using the same dataset as the one analysed here. We also highlight that the present approach, which carefully examines the acquisition of a particular morphological inflection in fine-grained detail, is useful for selecting areas of the Spanish inflectional system most predictive of DLD in developing assessments to identify atypical development in the context of US Spanish. Moreover, verbal agreement morphology, including in the preterit, is ubiquitous, so clinicians could assess children’s language samples to determine their degree of mastery of this structure. Low levels of verbal agreement, particularly with third person contexts, could be an indicator of the need for further testing.

Finally, our fourth research question targeted differences between groups in their patterns of non-target responses. We hypothesized that, given overall greater challenges with finiteness, children with DLD would produce a larger number of infinitives and bare verbs in place of inflected forms, while bilinguals would produce more substitutions of forms such as the tensed third person singular in place of other inflections. Our results were partially borne out. Descriptively, children across groups extended third person singular to all other contexts and also used the third person plural in first person plural contexts. Importantly, differences between the production of underspecified forms on the one hand and minimal bare verbs, gerunds, and infinitives on the other were not significant in the statistical modelling, contra the hypothesis. Moreover, children produced more gerunds than infinitives, which has not yet been documented.

We refer to children’s overextension of third person (both singular and plural) in the past tense as underspecified forms, indicating that certain grammatical features have been dropped. In first and second person singular contexts, children primarily produced the third person singular. In first person plural contexts, children substituted both third person singular and third person plural forms. This finding is compatible with the arguments of Borer and Rohrbacher (2002, pp. 159–160), who argue that children may be able to project some features consistently (i.e., number) without doing so with others (i.e., person). This asymmetry can result in underspecified forms emerging in some instances (i.e. first and second person or plural number) but not others (e.g. more consistent use of plural morphology than first or second person morphology). Finally, in third person plural contexts, children substituted third person singular forms.

These findings imply that children may drop person and, to a lesser degree, number, and instead might produce underspecified forms carrying third person and/or singular morphology. The use of underspecified forms therefore appears to be patterned, such that third person extends to first and second person contexts and singular number extends to plural contexts, but not vice versa. This finding aligns with extant research on heritage Spanish (Anderson, 2001; Camacho, 2022; Giancaspro & Higdon, 2024). However, we note that these patterns are largely consistent across groups, such that DLD or bilingualism does not result in qualitatively different tendencies, as had been predicted. Importantly, both underspecified and bare forms appear to co-occur here, so children may be in a protracted period of acquiring the ability to stably produce the appropriate person, number, and tense morphology, resulting in optionality. This optionality can take the form of target-like production, inflected defaults, or non-finite forms such as gerunds and bare verbs (and occasionally morphological infinitives).

Therefore, while we found both underspecification and bare verbs in children’s production tendencies, these patterns cannot serve as qualitative indicators of DLD because we do not find group-level differences in the distribution of non-target inflected versus uninflected forms in the statistical modelling. Verbal agreement is therefore a quantitative indicator of DLD in both bilingual and monolingual populations, as well as an inflectional structure that does not reach ceiling in typically developing children during the age range tested. However, non-target production tendencies do not appear to hold promise in identifying DLD, unlike in the case of clitic omissions in Thane et al. (2025) using the same dataset. Our findings are therefore compatible with previous work by Grinstead and colleagues (Grinstead et al., 2009a, 2009b, 2013; Pratt & Grinstead, 2007, 2008) that argue that third person singular forms in the present tense comprise bare verbs that do not contain inflectional morphology; this pattern co-occurs with the underspecification of person or number advanced by Borer and Rohrbacher (2002), perhaps even within the same children.

We would also like to note that these results differ subtly from Grinstead (2009b), who reported a pragmatic task effect where participants overproduced plural morphology in expectedly singular contexts since there were multiple characters in the pictures in the stimuli. In contrast, our data point to “oversingularization” in plural contexts as well as overextension of third person plural to first person plural contexts. We do not reproduce the effect of over pluralization that these authors found.

Before concluding, we wish to acknowledge the limitations of our study. Firstly, along with Grinstead et al. (2009b), we acknowledge that forced choice tasks allow for a broader overview of children’s holistic linguistic competence, and as such may be able to contribute to demonstrating whether children prefer third person singular or infinitival forms in place of grammatical alternatives. We recognize that future research would benefit from the inclusion of receptive data with which to tap comprehension of (lack of) person/number inflection. Similarly, we used elicited production data, which could result in lower levels of target-like production of verbal agreement than in spontaneous samples, as was the case when comparing the results of Bedore and Leonard’s (2001, 2005) elicited and spontaneous tasks. Future work triangulating elicited and spontaneous production data with a receptive task would be welcomed.

Another limitation of our study is its focus on group-level differences that do not take individual patterns of exposure into consideration (see Paradis, 2023 for discussion). While we have stated the case for considering bilingual variation in the recognition of DLD, we acknowledge that truly differentiating between atypical development and TD requires considering individual differences. Research has called for a “paradigm shift” (Giancaspro & Sánchez, 2021, p. 484) in HL acquisition to concentrate on individual-level patterns that move beyond group-level analyses. Future assessments of DLD in bilinguals must therefore consider individual environmental circumstances such as type of education (bilingual versus monolingual) and patterns of HL exposure because HSs who are exposed to or use Spanish less frequently have also been shown to produce lower levels of target-like verbal agreement than peers (Anderson, 2001; Goldin, 2022). In our study, there was considerable variation in the production of target-like verbal agreement within bilingual children, including those with TD. Moreover, there was an overlap between children with lower percentages of target-like performance in the BL-TD group and higher performers in the BL-DLD group. A possible explanation that urgently merits future research is that children who have typical language abilities but low exposure to Spanish could appear especially similar to children with DLD. Such trends cannot be captured in the group-level data available here, yet could explain the within-group variability, which has immediate implications for improving diagnostic accuracy.

## 8. Conclusion

Verbal agreement inflection in Spanish promises to be a very useful domain for disentangling typical and atypical HL development. The present study built upon extant research in multiple ways. Firstly, it compared bilingual and monolingual children with both typical and atypical development side-by-side. This approach contributes to a *disorder within difference* approach that characterizes the linguistic heterogeneity of US Spanish (Oetting, 2018; Oetting et al., 2016) while also elucidating structures that can be used to identify DLD through inflectional morphology in both bilingual and monolingual speakers. Secondly, our study is innovative in exploring the differences in substitution patterns employed in four populations of young Spanish-speaking children.

Summarizing the results of the present study, verbal agreement is a reliable predictor of DLD at the quantitative level because groups of children with TD produced more target-like preterit verbal agreement inflection than groups of peers with DLD, in each of the language populations (bilinguals and monolinguals). Importantly, the study showed significant differences between ML-DLD and BL-TD groups. This suggests that verbal agreement inflection is a promising domain to mitigate the overidentification of bilingual children. Finally, the BL-TD and ML-TD groups in our study did not significantly differ at the p < .05 level, arguing against robust bilingualism effects, at least within the bilingual population sampled in the present study. At the same time, the ML-DLD group produced significantly more target-like verbal agreement than BL-DLD peers. Regarding qualitative differences, that is, what children produce in place of target-like morphology, there is evidence of underspecified default forms for the third person singular and plural in the past tense. Importantly, children almost never extend the first person to non-first person contexts and never use the plural in singular ones. In addition, they produce bare verbs and gerunds, and therefore alternate between target-like, underspecified, and unspecified forms. All groups of children showed evidence of underspecified forms in place of non-target ones, so they do not appear to be qualitative indicators specific to DLD populations. Individual-level data would be useful for elaborating on the within-group variation observed here, as would a receptive forced choice task for better understanding children’s extension of third person forms alongside spontaneous production data.

## Footnotes

1 Some of these are portmanteau morphemes that simultaneously encode person and number, such as /o/ for the first person singular in the present tense.

2 We note, however, that articles are not as challenging to acquire as clitics in Thane et al. (2025). Therefore, other factors are also likely at play in mediating acquisition delays.

3 The DEME task was not originally designed to capture the source of possible differences between different person/number combinations using only preterit data. Second person plural was not elicited because it is homophonous with third person plural. We recognize that the inclusion of only one second person singular form in the preterit may have been a limitation of our study, particularly since most research has not considered it either.

4 Recall that any form not involving the present tense was coded as 3PS because it is possible to discern such forms from a bare verb (lexical root + thematic vowel) that is homophonous with the third person singular in the present simple tense. We refer to these substitutions as underspecified forms because they drop some, but not all, features such as person, number, or tense. In contrast, in alignment with Grinstead et al. (2013), we argue that verbs that on the surface appear to be third person singular in the present tense are in fact minimal bare forms and do not carry tense, person, or number.

## Long descriptions

### Table 1. Long description

The table is organized into five columns. The first column lists the Person and Number. The next two columns cover Infinitives ending in -ar, subdivided into Present simple and Preterit. The final two columns cover Infinitives ending in -er or -ir, also subdivided into Present simple and Preterit.

* First person singular: -ar verbs use -o (Present) and -é (Preterit); -er/ir verbs use -o (Present) and -í (Preterit).

* First person plural: -ar verbs use -amos for both tenses; -er/ir verbs use -emos or -imos (Present) and -imos (Preterit).

* Second person singular: -ar verbs use -as (Present) and -aste (Preterit); -er/ir verbs use -es (Present) and -iste (Preterit).

* Second person plural: -ar verbs use -an (Present) and -aron (Preterit); -er/ir verbs use -en (Present) and -ieron (Preterit).

* Third person singular: -ar verbs use -a (Present) and -ó (Preterit); -er/ir verbs use -e (Present) and -ió (Preterit).

* Third person plural: -ar verbs use -an (Present) and -aron (Preterit); -er/ir verbs use -en (Present) and -ieron (Preterit).

Navigate back to Table 1..

### Figure 1. Long description

The chart consists of two panels with a shared y-axis labeled Percentage of target responses ranging from 0 to 100. The x-axis for both panels lists four groups: M L dash T D, M L dash D L D, B L dash T D, and B L dash D L D.

Left Panel: Average S D by group. This bar graph shows the mean percentage with standard deviation in parentheses above each bar.

- M L dash T D: yellow bar at 78 with S D 17.

- M L dash D L D: light blue bar at 51 with S D 23.

- B L dash T D: pink bar at 68 with S D 22.

- B L dash D L D: teal bar at 27 with S D 19.

Right Panel: Distribution by group. This box plot shows the median, quartiles, and outliers for the same groups.

- M L dash T D: yellow box with a median around 72 and one low outlier.

- M L dash D L D: light blue box with a median around 58 and three outliers near 15 and 0.

- B L dash T D: pink box with a median around 72 and one outlier at 0.

- B L dash D L D: teal box with a median around 28 and no visible outliers.

Navigate back to Figure 1..

### Table 2. Long description

The table contains six columns: Variable, Estimate, Probability, S E, p, and Reference group. It is divided into four sections based on the Reference group.

1. Reference group M L dash T D:

- Intercept (M L dash T D): Estimate 1.40, Probability 80.2%, S E 0.42, p < .001.

- M L dash D L D: Estimate -1.40, Probability 50.1%, S E 0.32, p < .001.

- B L dash T D: Estimate -0.53, Probability 70.5%, S E 0.31, p .085.

- B L dash D L D: Estimate -2.34, Probability 28.1%, S E 0.33, p < .001.

- Age in months: Estimate 0.05, Probability 88.0%, S E 0.01, p < .001.

2. Reference group M L dash D L D:

- Intercept (M L dash D L D): Estimate 0.00, Probability 50.1%, S E 0.40, p .994.

- M L dash T D: Estimate 1.40, Probability 80.2%, S E 0.32, p < .001.

- B L dash T D: Estimate 0.87, Probability 70.5%, S E 0.28, p .002.

- B L dash D L D: Estimate -0.94, Probability 28.1%, S E 0.30, p .002.

- Age in months: Estimate 0.05, Probability 64.4%, S E 0.01, p < .001.

3. Reference group B L dash T D:

- Intercept (B L dash T D): Estimate 0.87, Probability 70.5%, S E 0.39, p .026.

- M L dash T D: Estimate 0.53, Probability 80.2%, S E 0.31, p .085.

- M L dash D L D: Estimate -0.87, Probability 50.1%, S E 0.28, p .002.

- B L dash D L D: Estimate -1.81, Probability 28.1%, S E 0.29, p < .001.

- Age in months: Estimate 0.05, Probability 81.2%, S E 0.01, p < .001.

4. Reference group B L dash D L D:

- Intercept (B L dash D L D): Estimate -0.94, Probability 28.1%, S E 0.40, p .019.

- M L dash T D: Estimate 2.34, Probability 80.2%, S E 0.33, p < .001.

- M L dash D L D: Estimate 0.94, Probability 50.1%, S E 0.30, p .002.

- B L dash T D: Estimate 1.81, Probability 70.5%, S E 0.29, p < .001.

- Age in months: Estimate 0.05, Probability 41.4%, S E 0.01, p < .001.

Navigate back to Table 2..

### Table 3. Long description

The table consists of four columns: Contrast, Estimate, S E, and p.

* Row 1: (M L - T D) minus (M L - D L D) has an estimate of 1.40, S E of 0.32, and p less than .001.

* Row 2: (M L - T D) minus (B L - T D) has an estimate of 0.53, S E of 0.31, and p of .311.

* Row 3: (M L - T D) minus (B L - D L D) has an estimate of 2.34, S E of 0.33, and p less than .001.

* Row 4: (M L - D L D) minus (B L - T D) has an estimate of minus 0.87, S E of 0.28, and p of .012.

* Row 5: (M L - D L D) minus (B L - D L D) has an estimate of 0.94, S E of 0.30, and p of .009.

* Row 6: (B L - T D) minus (B L - D L D) has an estimate of 1.81, S E of 0.29, and p less than .001.

Navigate back to Table 3..

### Table 4. Long description

The table categorizes non-target responses into ten types across four groups. Each group has a count (#) and percentage (%) column.

* Stem: M L-T D (1, 2.6%), M L-D L D (0, 0.0%), B L-T D (1, 1.3%), B L-D L D (1, 0.6%), Total 3.

* Unintelligible: M L-T D (2, 5.3%), M L-D L D (27, 30.7%), B L-T D (7, 9.3%), B L-D L D (51, 30.4%), Total 87.

* Unrelated/No S P A verb: M L-T D (12, 31.6%), M L-D L D (11, 12.5%), B L-T D (5, 6.7%), B L-D L D (14, 8.3%), Total 42.

* 1 P S: M L-T D (0, 0.0%), M L-D L D (1, 1.1%), B L-T D (1, 1.3%), B L-D L D (3, 1.8%), Total 5.

* 3 P S: M L-T D (2, 5.3%), M L-D L D (12, 13.6%), B L-T D (20, 26.7%), B L-D L D (30, 17.9%), Total 64.

* 3 P P: M L-T D (8, 21.1%), M L-D L D (12, 13.6%), B L-T D (13, 17.3%), B L-D L D (12, 7.1%), Total 45.

* Bare: M L-T D (1, 2.6%), M L-D L D (9, 10.2%), B L-T D (8, 10.7%), B L-D L D (22, 13.1%), Total 40.

* Gerund: M L-T D (3, 7.9%), M L-D L D (2, 2.3%), B L-T D (6, 8.0%), B L-D L D (15, 8.9%), Total 26.

* Infinitive: M L-T D (0, 0.0%), M L-D L D (1, 1.1%), B L-T D (2, 2.7%), B L-D L D (8, 4.8%), Total 11.

* Non-target tense: M L-T D (9, 23.7%), M L-D L D (13, 14.8%), B L-T D (12, 16.0%), B L-D L D (12, 7.1%), Total 46.

* Total: M L-T D (38, 100%), M L-D L D (88, 100%), B L-T D (75, 100%), B L-D L D (168, 100%), Grand Total 369.

Navigate back to Table 4..

### Figure 2. Long description

The x-axis is labeled Group and contains four categories: M L dash T D, M L dash D L D, B L dash T D, and B L dash D L D. The y-axis is labeled Number forward slash distribution of non-target responses, ranging from 0 to over 150. A legend on the right identifies ten response types: 1 P S, 3 P S, 3 P P, Bare, Gerund, Infinitive, Non-target tense, Stem, Unintelligible, and Unrelated forward slash no S P A verb.

* M L dash T D has the lowest total count, under 50, dominated by Unrelated forward slash no S P A verb and 3 P P.

* M L dash D L D has a total count near 90, with large segments for Unintelligible, Non-target tense, and 3 P S.

* B L dash T D has a total count near 75, with 3 P S and 3 P P being the largest segments.

* B L dash D L D has the highest total count, exceeding 150. The largest segments are Unintelligible, 3 P S, and Gerund.

Across all groups, Unintelligible and 3 P S are consistently prominent non-target response types.

Navigate back to Figure 2..

### Table 5. Long description

The table contains six columns: Variable, Estimate, Probability, S E, p, and Model. It is divided into two primary models.

1. Language ability model:

- Intercept T D: Estimate 1.33, Probability 79.0 percent, S E 0.31, p less than 0.001.

- D L D: Estimate minus 0.82, Probability 62.4 percent, S E 0.34, p .017.

2. Bilingualism model:

- Intercept Monolinguals: Estimate 1.00, Probability 73.1 percent, S E 0.25, p less than 0.001.

- Bilinguals: Estimate minus 0.59, Probability 60.2 percent, S E 0.33, p .077.

Navigate back to Table 5..

### Figure 3. Long description

The chart consists of five stacked horizontal panels, each representing an ‘Expected Inflection’: 1 P S, 1 P P, 2 P S, 3 P S, and 3 P P. The y-axis for all panels is ‘Number of substitutions per group’ ranging from 0 to 15. The x-axis lists four groups: M L T D, M L D L D, B L T D, and B L D L D. A legend on the right identifies ‘Form produced’ by color: 1 P S (dark blue), 3 P S (light blue), 3 P P (green), Bare (orange), Gerund (purple), and Infinitive (yellow).

* Expected 1 P S panel: M L T D has 1 3 P S. M L D L D has 1 1 P S, 6 3 P S, and 3 Bare. B L T D has 9 3 P S and 1 Bare. B L D L D has 8 3 P S, 6 Bare, 4 Gerund, and 1 Infinitive.

* Expected 1 P P panel: M L T D has 8 3 P P and 3 Gerund. M L D L D has 11 3 P P, 4 Bare, and 2 Gerund. B L T D has 13 3 P P, 4 Bare, and 4 Gerund. B L D L D has 2 3 P S, 11 3 P P, 11 Bare, 5 Gerund, and 3 Infinitive.

* Expected 2 P S panel: M L T D has 1 3 P S and 1 Bare. M L D L D has 2 3 P S, 1 3 P P, and 1 Bare. B L T D has 8 3 P S and 1 Bare. B L D L D has 11 3 P S and 3 Bare.

* Expected 3 P S panel: M L D L D has 1 Infinitive. B L T D has 1 1 P S, 1 Bare, 1 Gerund, and 2 Infinitive. B L D L D has 3 1 P S, 5 Gerund, and 2 Infinitive.

* Expected 3 P P panel: M L D L D has 4 3 P S and 1 Bare. B L T D has 3 3 P S, 1 Bare, and 1 Gerund. B L D L D has 9 3 P S, 1 3 P P, 2 Bare, 1 Gerund, and 2 Infinitive.

Navigate back to Figure 3..

### Table 6. Long description

The table consists of six columns: Variable, Estimate, Probability, S E, p, and Model. It is divided into two primary models.

1. Language ability model:

- Intercept T D: Estimate 0.15, Probability 53.7 percent, S E 0.48, p-value .758.

- D L D: Estimate 0.91, Probability 74.3 percent, S E 0.50, p-value .066.

2. Bilingualism model:

- Intercept Monolinguals: Estimate 0.26, Probability 56.4 percent, S E 0.46, p-value .580.

- Bilinguals: Estimate 0.97, Probability 77.4 percent, S E 0.59, p-value .098.

Navigate back to Table 6..

### Figure 4. Long description

The x-axis is labeled Age in months, ranging from 48 to 84. The y-axis is labeled Percentage of target responses, ranging from 0 to 100. A legend on the right identifies four groups: M L dash T D in yellow, M L dash D L D in light blue, B L dash T D in red, and B L dash D L D in dark teal.

Individual data points are plotted as colored circles corresponding to these groups. Large overlapping ellipses represent the distribution for each group.

* The M L dash T D yellow ellipse is positioned highest, centered around 100 percent target responses between 60 and 84 months.

* The B L dash T D red ellipse is slightly lower and wider, showing a positive correlation between age and performance.

* The M L dash D L D light blue ellipse covers a broad vertical range from 0 to 100 percent, centered around 50 percent.

* The B L dash D L D dark teal ellipse is the lowest and smallest, concentrated between 48 and 72 months with most data points falling below 60 percent target responses.

Navigate back to Figure 4..
